# Role of Autophagy in Von Willebrand Factor Secretion by Endothelial Cells and in the In Vivo Thrombin-Antithrombin Complex Formation Promoted by the HIV-1 Matrix Protein p17

**DOI:** 10.3390/ijms21062022

**Published:** 2020-03-16

**Authors:** Antonella Bugatti, Stefania Marsico, Pietro Mazzuca, Kai Schulze, Thomas Ebensen, Cinzia Giagulli, Esther Peña, Lina Badimón, Mark Slevin, Arnaldo Caruso, Carlos A. Guzman, Francesca Caccuri

**Affiliations:** 1 Department of Molecular and Translational Medicine, Section of Microbiology, University of Brescia Medical School, 25123 Brescia, Italy; antonella.bugatti@unibs.it (A.B.); p.mazzuca@unibs.it (P.M.); cinzia.giagulli@unibs.it (C.G.); arnaldo.caruso@unibs.it (A.C.); 2 Department of Pharmacy, Health and Nutritional Sciences, University of Calabria, 87036 Arcavacata di Rende, Italy; stefania.marsico@unical.it; 3 Helmholtz Center for Infection Research (HZI), Department of Vaccinology and Applied Microbiology, 38124 Braunschweig, Germany; kai.schulze@helmholtz-hzi.de (K.S.); thomas.ebensen@helmholtz-hzi.de (T.E.); CarlosAlberto.Guzman@helmholtz-hzi.de (C.A.G.); 4 Cardiovascular Program ICCC, CiberCV, Hospital de la Santa Creu i Sant Pau, 08041 Barcelona, Spain; epena@santpau.cat (E.P.); lbadimon@santpau.cat (L.B.); 5 John Dalton Building, School of Healthcare Science, Manchester Metropolitan University, Manchester M1 5GD, UK; m.a.slevin@mmu.ac.uk

**Keywords:** HIV-1, p17 matrix protein, coagulation, thrombin-antithrombin complex, AIDS-related diseases, autophagy

## Abstract

Although the advent of combined antiretroviral therapy has substantially improved the survival of HIV-1-infected individuals, non-AIDS-related diseases are becoming increasingly prevalent in HIV-1-infected patients. Persistent abnormalities in coagulation appear to contribute to excess risk for a broad spectrum of non-AIDS defining complications. Alterations in coagulation biology in the context of HIV infection seem to be largely a consequence of a chronically inflammatory microenvironment leading to endothelial cell (EC) dysfunction. A possible direct role of HIV-1 proteins in sustaining EC dysfunction has been postulated but not yet investigated. The HIV-1 matrix protein p17 (p17) is secreted from HIV-1-infected cells and is known to sustain inflammatory processes by activating ECs. The aim of this study was to investigate the possibility that p17-driven stimulation of human ECs is associated with increased production of critical coagulation factors. Here we show the involvement of autophagy in the p17-induced accumulation and secretion of von Willebrand factor (vWF) by ECs. In vivo experiments confirmed the capability of p17 to exert a potent pro-coagulant activity soon after its intravenous administration.

## 1. Introduction

Autophagy has been largely known as a degradative process serving a pro-survival, metabolite-generating role. Over the last few decades, a continuous growing body of evidence points to a function of autophagy in secretion of different molecules under normal and pathological conditions. Enhancement or down-modulation of the autophagic molecular machinery is followed by alterations in secretion. Autophagy plays a critical role in regulating unconventional [1,2,3] and conventional [4] secretory pathways, whereas a growing number of studies have also recently suggested a connection between autophagy and secretion of intracellular granules [5,6].

Von Willebrand factor (vWF) is a glycoprotein stored in specific secretory granules known as Weibel-Palade bodies (WPBs) [7,8]. This molecule is contained mainly in endothelial cells (ECs) and once secreted it multimerizes and assembles into long strings which tether to the underlying connective tissue, playing a crucial role in platelet trapping to maintain normal homeostasis [9]. Reduced secretion of vWF results in bleeding disorders [10], whereas increased release of vWF following EC dysfunction is known to contribute to thrombotic disorders [11,12]. Autophagy has been recently shown to regulate EC processing, maturation and secretion of vWF [13]. Inhibition of the autophagic flux in ECs with chloroquine resulted in a marked down-modulation of vWF secretion, and chloroquine administration in mice resulted in an increased bleeding time [13]. These findings demonstrated, for the first time, that transient pharmacological inhibition of autophagic flux may be a useful strategy to prevent thrombotic events [13].

Combined antiretroviral therapy (cART) has dramatically improved the survival of human immunodeficiency virus type 1-infected (HIV^+^) patients. With control of HIV-1 replication, serious non-AIDS-related diseases are now the dominant cause of morbidity and mortality in cART-treated populations [14]. Particularly, cardiovascular diseases have emerged as a clinically significant issue [15]. Several reports indicate that HIV^+^ patients have an increased risk of venous thrombosis [16] and a greater risk for myocardial infarction [15] than uninfected control individuals. Abnormal levels of coagulation markers are observed in HIV^+^ individuals [17]. Mainly, elevated levels of vWF have been reported in patients with HIV-1 disease compared to levels in control individuals [18], whilst the administration of cART improves but does not fully normalize the level of this pro-coagulant molecule [19]. The precise mechanisms driving a state of activation of coagulation in HIV^+^ patients are not entirely clear. The evidence that successfully cART-treated patients with negative viremia display a hypercoagulable state suggests diverse pathogenic mechanisms at work in addition to the direct effects of the HIV-1 itself.

HIV-1-encoded proteins are expressed in HIV^+^ patients in the absence of viremia and they induce a chronically inflamed microenvironment leading to EC dysfunction [20]. In particular, the viral matrix protein p17 (p17) is detected in the blood [21] and accumulates in different tissues and organs [22,23,24], even in patients under successful cART [25,26]. This is not surprising since latently HIV-1-infected resting T cells are capable of producing *gag* proteins in a model of HIV-1 latency [27]. Moreover, recent data show that p17 is continuously released into the extracellular space from HIV-1-infected cells even in the absence of viral protease, following its cellular aspartyl protease-dependent cleavage from the *gag* precursor protein [28].

Extracellularly, p17 plays a critical role in the immune cell-mediated inflammatory processes [29,30,31] and it is known to activate ECs and promote a potent angiogenic activity [26,32]. Interestingly, we demonstrated that angiogenesis induced by p17 is partly supported by the release of ET-1 and by activation of the ET-1/ET-1 B receptor axis [32]. ET-1 secretion from ECs upon p17 stimulation was found to rely on mechanisms of conventional secretory pathways regulated by autophagy both in vitro and in vivo [33].

In this study, we provide evidence that the p17-driven activation of human ECs is associated with an increased cytoplasmic accumulation and secretion of vWF following activation of the autophagy pathway. Moreover, the intravenous (i.v.) injection of p17 promotes a pro-coagulant state in vivo, which does not occur in autophagy-deficient animals. These findings offer a new way of thinking about the possible cause of increased risk for coagulopathy in people living with HIV-1 and suggest autophagy as a specific target for treating or preventing coagulation disorders.

## 2. Results

### 2.1. The HIV-1 Matrix Protein p17 Induces vWF Cytoplasmic Accumulation in ECs

**Figure 1:**
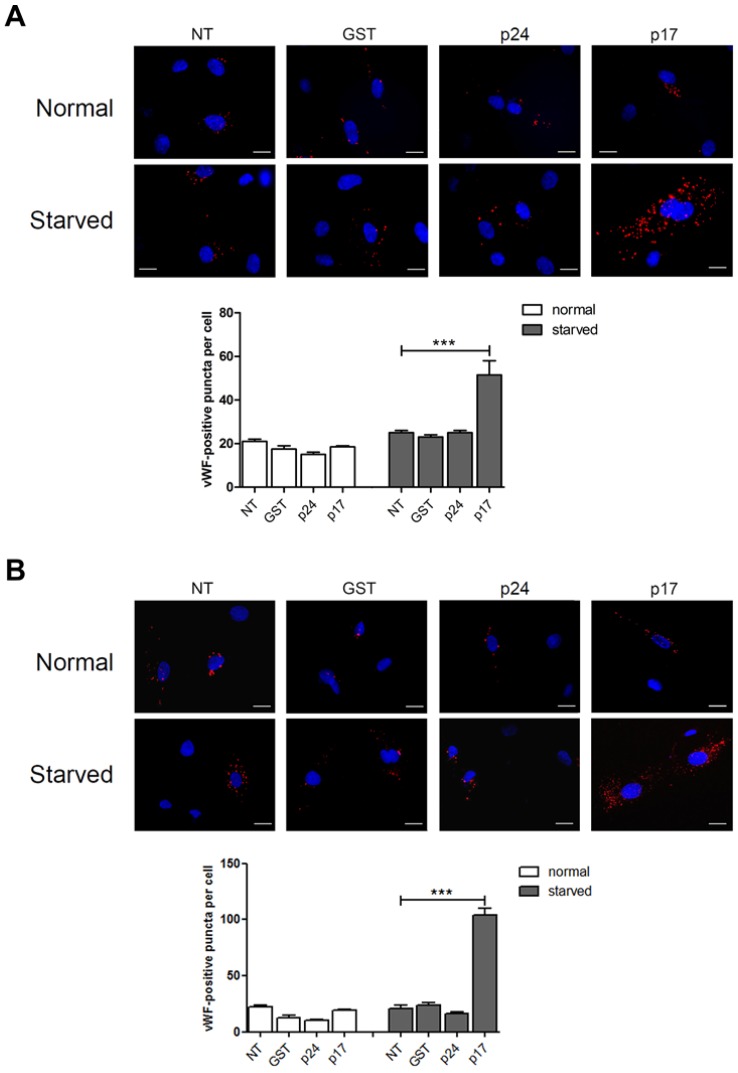
The HIV-1 matrix protein p17 induces von Willebrand factor (vWF) accumulation in Weibel-Palade bodies (WPBs) under serum deprivation. HUVECs (**A**) and HMVEC-Ls (**B**) were nucleofected with a mCherry-vWF-expressing plasmid and 24 h after nucleofection cells were starved or not for 16 h and then stimulated in the presence or absence of 10 ng/mL of GST, p24 or p17 in complete medium. The images display vWF signals in red and cell nuclei in blue. Scale bar, 10 μm. Red-positive punctate structures were counted in order to quantify the levels of WPBs. NT indicates not treated cells. Values reported for vWF positive structures are the mean ± SD of 3 independent experiments with similar results. Statistical analysis was performed by one-way ANOVA, and the Bonferroni post-test was used to compare data (*** *p* < 0.001).

In order to understand the role of p17 in vWF accumulation and secretion, a mCherry-vWF-expressing plasmid was used to transfect human umbilical vein endothelial cells (HUVECs) and monitor vWF accumulation in WPBs by the classical red bright cigar-shaped appearance in the cytoplasm [13]. Nucleofected HUVECs were then cultured under normal or serum-deprived conditions in the presence or absence of p17 (Figure 1A). Under normal culture conditions, p17 did not increase WPBs accumulation of vWF as compared to untreated (NT) cells or to cells treated with the irrelevant protein GST or the HIV-1 capsid protein p24 (p24) (Figure 1A). In contrast, serum starved HUVECs showed an increased accumulation of vWF in response to p17 stimulation as compared to NT cells or to cells treated with GST or p24 (Figure 1A and Supplementary Figure S1). The effect of p17 on vWF accumulation observed in macrovascular ECs was also confirmed in microvascular ECs using the human lung microvascular endothelial cells (HMVEC-Ls) model (Figure 1B).

**Figure 2:**
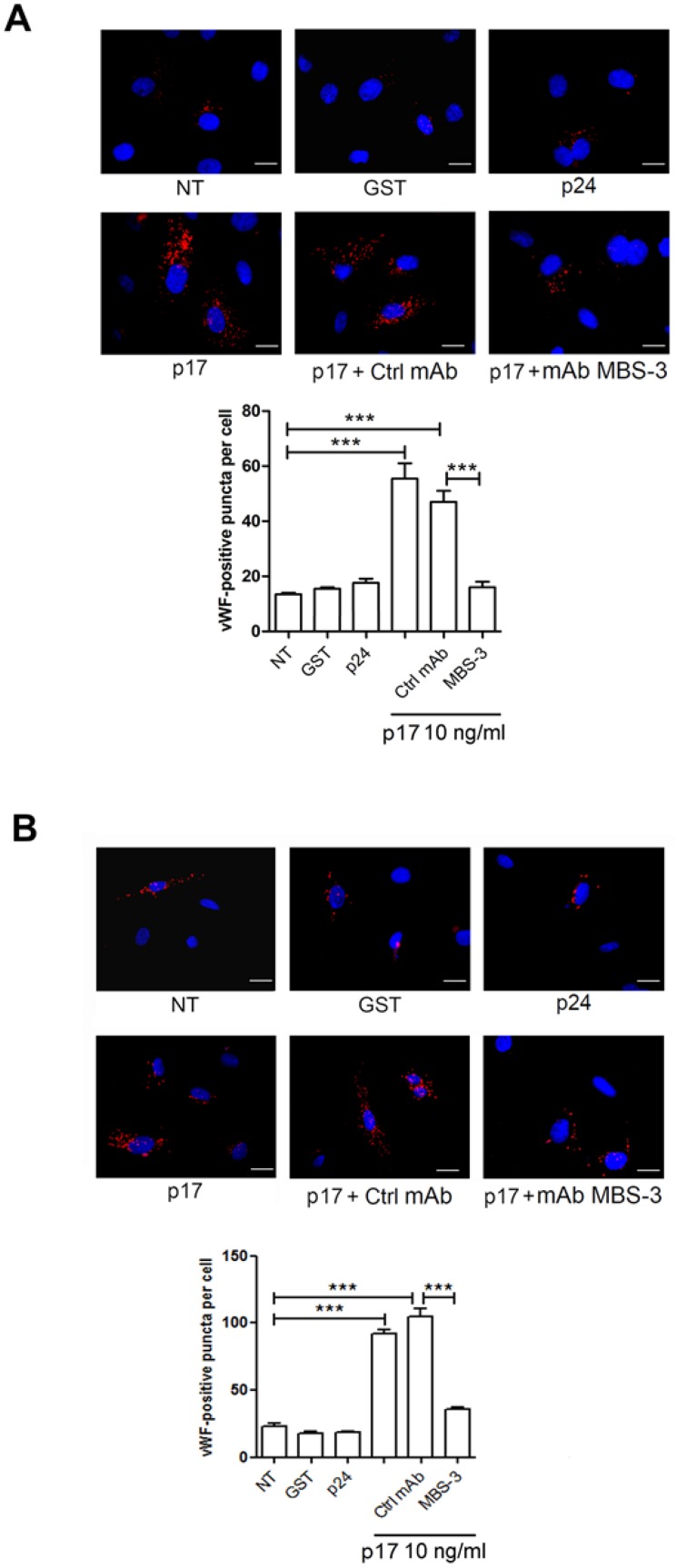
vWF accumulation in WPBs is specifically induced by p17. mCherry-vWF nucleofected HUVECs (**A**) and HMVEC-Ls (**B**) were serum starved for 16 h and then treated with 10 ng/mL of GST, p24 or p17 in complete medium. When indicated, cells were stimulated with p17 (10 ng/mL) after preincubation of the viral protein with 1 µg/mL of mAb to p17 (MBS-3) or unrelated control mAb (Ctrl mAb) for 30 min at 37 °C. The images display vWF signals in red and cell nuclei in blue. Scale bar, 10 μm. Red-positive punctate structures were counted in order to quantify the levels of WPBs. NT indicates not treated cells. Values reported for vWF positive structures are the mean ± SD of 3 independent experiments with similar results. Statistical analysis was performed by one-way ANOVA, and the Bonferroni post-test was used to compare data (*** *p* < 0.001).

The effect of p17 was abrogated by preincubating the medium containing p17 with the p17 mAb MBS-3, thus confirming the specificity of p17 activity both in HUVEC (Figure 2A) and in HMVEC-Ls (Figure 2B). Altogether, our data demonstrate that cytoplasmic vWF accumulation upon p17 treatment is specific and depends on activation of a cellular stress pathway.

### 2.2. Autophagy Controls the Storage and Secretion of vWF Upon p17 Stimulation

**Figure 3:**
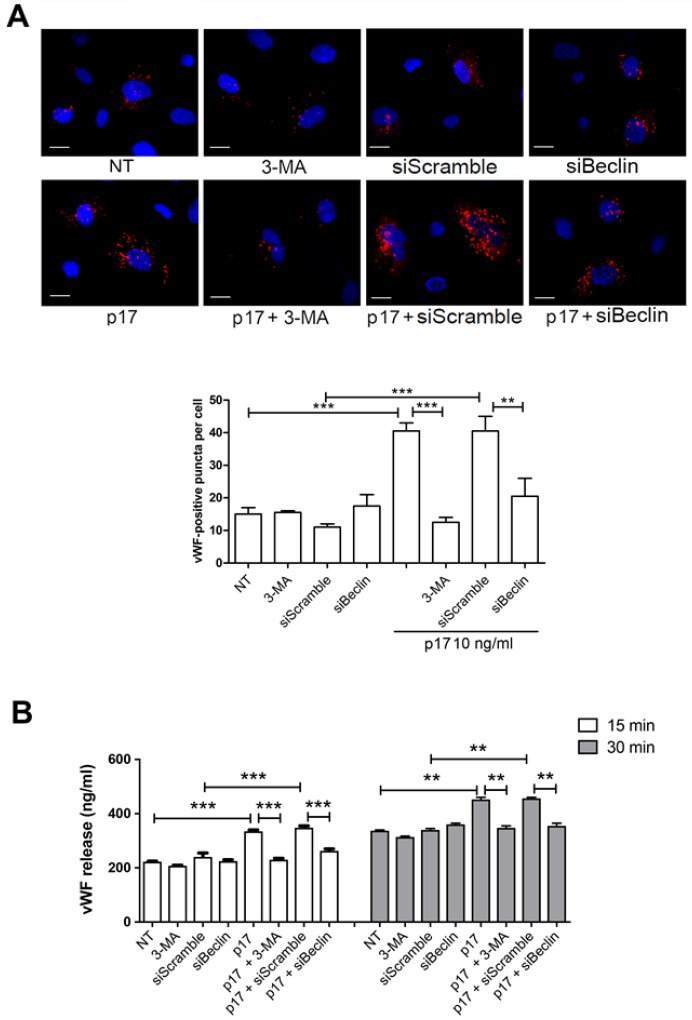
Inhibition of autophagy decreases p17-mediated release of vWF. (**A**) HUVECs were nucleofected with a mCherry-vWF-expressing plasmid and, when indicated, in combination with siBeclin-1 or siScramble. Twenty-four h after nucleofection, cells were serum starved for 16 h and then stimulated or not with 10 ng/mL of p17 in complete medium. When indicated, HUVECs were serum starved for 16 h in the presence or absence of 3-MA (5 mM). The images display vWF signals in red and cell nuclei in blue. Scale bar, 10 μm. Red-positive punctate structures were counted in order to quantify the levels of WPBs. NT indicates not treated cells. Values reported for vWF positive structures are the mean ± SD of 3 independent experiments with similar results. Statistical analysis was performed by one-way ANOVA, and the Bonferroni post-test was used to compare data (** *p* < 0.01, *** *p* < 0.001). (**B**) HUVECs were serum starved for 16 h in the presence or absence of 3-MA (5 mM). When indicated, cells were nucleofected with siBeclin-1 or siScramble and serum starved 24 h after nucleofection. After serum starvation, cells were cultured in complete medium containing or not 10 ng/mL of p17. Supernatants were collected at 15 and 30 min of culture and analyzed for the presence of vWF by a standard quantitative ELISA. Bars represent the mean ± SD of triplicate samples. Statistical analysis was performed by one-way ANOVA, and Bonferroni post-test was used to compare data (** *p* < 0.01, *** *p* < 0.001). NT indicates not treated cells.

In addition to the well-known mechanism of vWF secretion, which is dependent on the classical endoplasmic reticulum (ER)–Golgi complex pathway, autophagy plays a critical role in the biology of WPBs and regulates the in vitro and in vivo release of vWF [13]. Recently, p17-mediated lymphangiogenesis was found to depend on secretory autophagy pathway and secretion of ET-1 by ECs [32,33]. Thus, we tested the ability of p17 to promote vWF storage and secretion from endothelium and the involvement of autophagy in its activity. We used 3-MA, a synthetic and cell-permeable autophagic sequestration blocker [34] to pharmacologically inhibit autophagy. Nucleofected HUVECs with a mCherry-vWF-expressing plasmid were serum starved for 16 h in the presence or absence of 3-MA and then incubated or not with p17. Inhibition of autophagy with 3-MA significantly decreased the vWF cytoplasmic storage induced by p17 stimulation (Figure 3A). To further confirm the involvement of autophagy in the p17 capability to promote vWF accumulation in WPBs, we used siRNA to inhibit the expression of Beclin-1 (siBeclin), a coiled-coil protein involved in the regulation of autophagy in mammalian cells [35]. HUVECs were co-nucleofected with the mCherry-vWF-expressing plasmid and siBeclin. SiScramble was used as a negative control. Then, 24 h after nucleofection, cells were serum starved for 16 h and then stimulated or not with p17. As expected, inhibition of autophagy by Beclin-1 gene silencing significantly decreased the p17-triggered vWF accumulation in WPBs (Figure 3A). To better characterize the capability of p17 to trigger secretion of constitutively produced vWF, we performed a kinetic study by collecting supernatants of serum-starved HUVECs for 16 h in the presence or absence of 3-MA that were then cultured for 15 and 30 min in complete medium containing or not p17. Kinetic studies were also run using serum-starved HUVECs silenced or not with siBeclin 24 h before serum starvation. As shown in Figure 3B, p17 induced a significant increase of vWF secretion at 15 and 30 min of HUVECs stimulation, as compared to untreated cells. At the same time, p17-triggered vWF secretion was significantly inhibited by silencing of Beclin-1 or by pretreatment of HUVECs with 3-MA.

**Figure 4:**
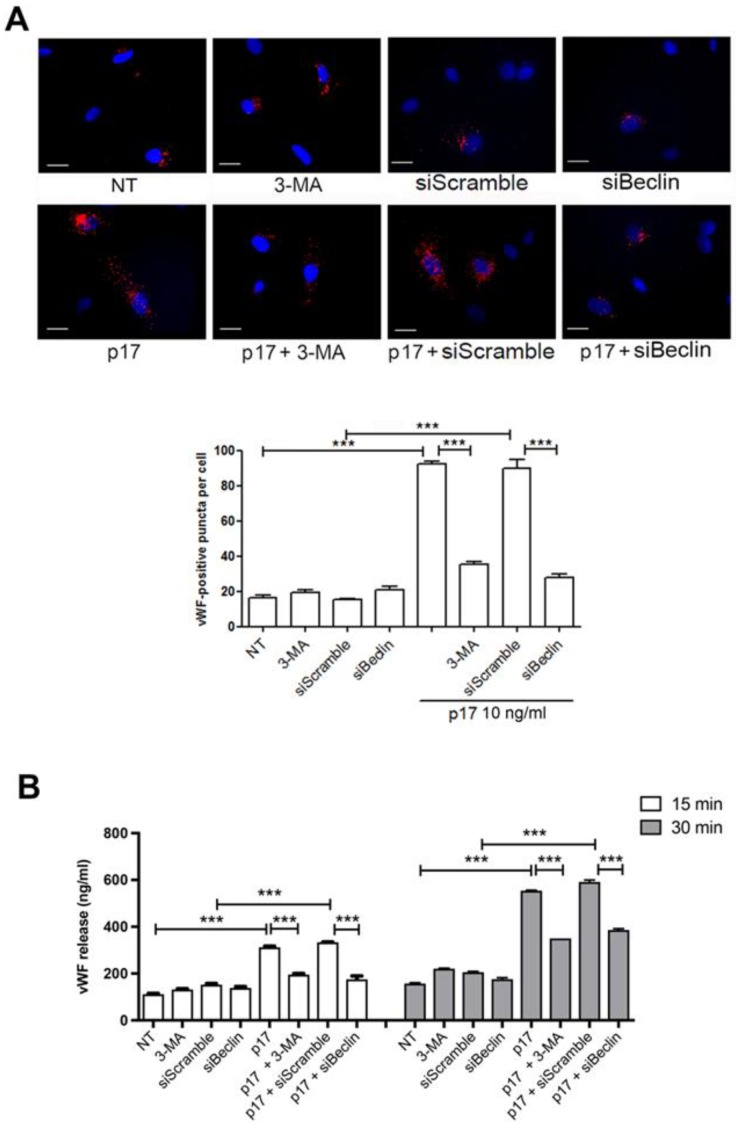
Inhibition of autophagy decreases p17-mediated release of vWF in HMVEC-Ls. (**A**) HMVEC-Ls were nucleofected with a mCherry-vWF plasmid and, when indicated, in combination with siBeclin-1 or irrelevant (siScramble) siRNAs. Twenty-four h after nucleofection, cells were serum starved for 16 h and then stimulated or not with 10 ng/mL of p17 in complete medium. When indicated, HMVEC-Ls were serum starved for 16 h in the presence or absence of 3-MA (5 mM). The images display vWF signals in red and cell nuclei in blue. Scale bar, 10 μm. Red-positive punctate structures were counted in order to quantify the levels of WPBs. NT indicates not treated cells. Values reported for vWF positive structures are the mean ± SD of 3 independent experiments with similar results. Statistical analysis was performed by one-way ANOVA, and the Bonferroni post-test was used to compare data (*** *p* < 0.001). (**B**) HMVEC-Ls were serum starved for 16 h in the presence or absence of 3-MA (5 mM). When indicated, cells were nucleofected with siBeclin-1 or irrelevant (siScramble) siRNAs and 24 h after nucleofection were serum starved. After serum starvation, cells were cultured in complete medium containing or not 10 ng/mL of p17. Supernatants were collected at 15 and 30 min of culture and analyzed for the presence of vWF by a standard quantitative ELISA. Bars represent the mean ± SD of triplicate samples. Statistical analysis was performed by one-way ANOVA, and Bonferroni post-test was used to compare data (*** *p* < 0.001). NT indicates not treated cells.

The role of autophagy in the intracellular vWF accumulation and its secretion following p17 stimulation was further confirmed in HMVEC-Ls (Figure 4A,B). These data demonstrate that vWF secretion in p17-treated ECs is dependent on the activation of an autophagy-based pathway.

### 2.3. Mechanistic Insight in the p17-Triggered Processing of vWF

**Figure 5:**
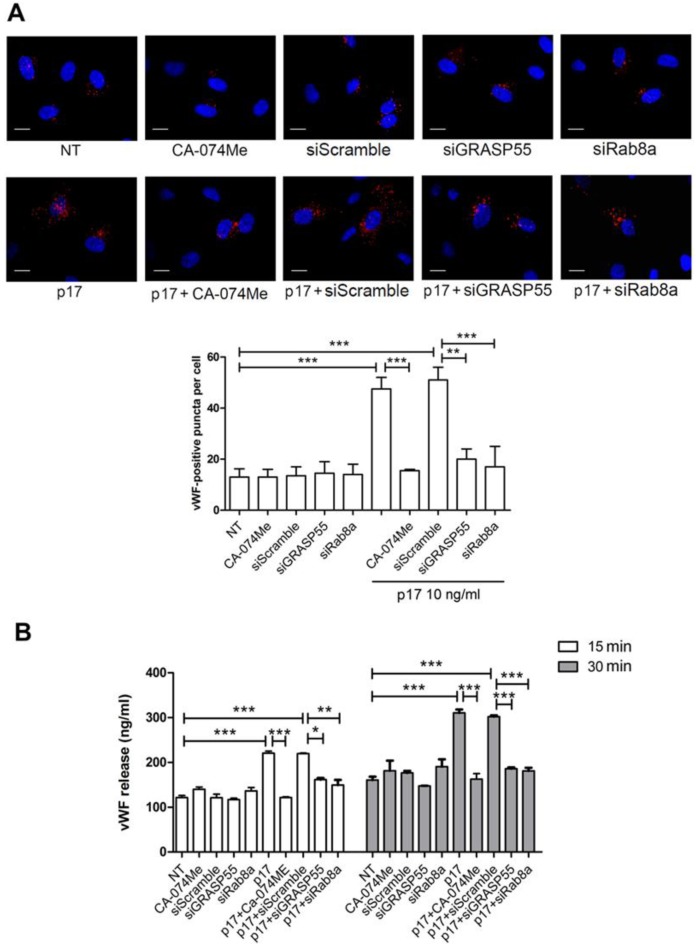
Mechanistic insights into the vWF secretory activity of p17. (**A**) HUVECs were nucleofected with a mCherry-vWF-expressing plasmid in combination or not with siGRASP55, siRab8a or siScramble. Twenty-four h after nucleofection, cells were serum starved for 16 h and then treated with 10 ng/mL of p17. When reported, HUVECs were serum starved in the presence or absence of 50 µM of CA-074Me (cathepsin-β inhibitor) and then stimulated or not with 10 ng/mL of p17. The images display vWF signals in red and cell nuclei in blue. Scale bar, 10 μm. Red-positive punctate structures were counted in order to quantify the levels of WPBs. NT indicates not treated cells. Values reported for vWF positive structures are the mean ± SD of 3 independent experiments with similar results. Statistical analysis was performed by one-way ANOVA, and the Bonferroni post-test was used to compare data (** *p* < 0.01, *** *p* < 0.001). (**B**) HUVECs were serum starved in the presence or absence of 50 μM of CA-074Me. In some experiments, HUVECs were nucleofected with siScramble, siGRASP55 or siRab8a and 24 h after nucleofection, cells were serum starved for 16 h. After serum starvation, HUVECs were cultured in complete medium containing or not 10 ng/mL of p17. After 15 and 30 min of culture, supernatants were collected and analyzed by a standard quantitative ELISA. Bars represent the mean ± SD of triplicate samples. Statistical analysis was performed by one-way ANOVA, and the Bonferroni post-test was used to compare data (* *p* < 0.05, ** *p* < 0.01, *** *p* < 0.001). NT indicates not treated cells.

The secretion of proteins by the autophagy-based process without entering the classical ER–Golgi complex pathway is now established in eukaryotic cells [1,3,36]. Hence, to better understand the role of autophagy in p17-mediated vWF secretion from HUVECs, we silenced two crucial genes involved in autophagy, namely GRASP55 [3,36] and Rab8a [3]. Silencing of each of these genes in serum-starved HUVECs was sufficient to reduce WBPs storage of vWF promoted by p17 stimulation (Figure 5A). As expected, no effect on vWF accumulation was observed in silenced GRASP55 and Rab8a HUVECs in the absence of p17 stimulation. Since the autophagy-driven secretion of proteins and cytokines requires cathepsin-β activation [3], we pharmacologically inhibited cathepsin-β through the specific inhibitor CA-074Me. This blockage normalized p17-driven intracellular accumulation of vWF. Moreover, inhibition of GRASP55, Rab8a and cathepsin-β activity resulted in suppression of p17-induced vWF secretion (Figure 5B).

Altogether, our data support the key role played by autophagy in the p17-triggered vWF secretion.

### 2.4. P17-Triggered vWF Storage and Secretion Requires Activation of PI3K

**Figure 6:**
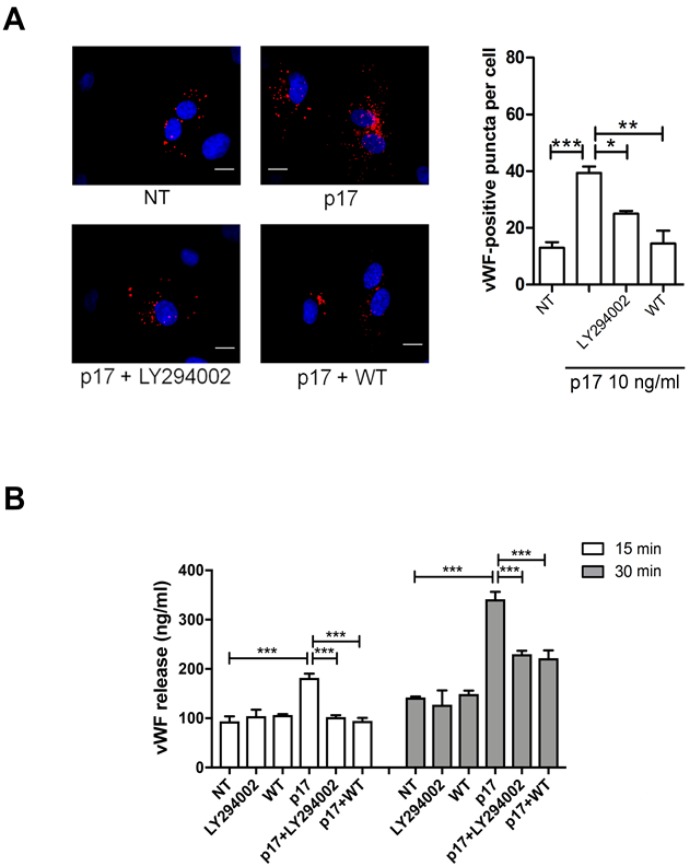
Role of PI3K in p17-induced vWF secretion. (**A**) HUVECs were nucleofected with a mCherry-vWF-expressing plasmid. Twenty-four h after nucleofection, cells were serum starved for 16 h and then treated for 1 h with the PI3K inhibitor LY294002 (10 μM) or wortmannin (WT [100 nM]) before stimulation with p17 (10 ng/mL). The images display vWF signals in red and cell nuclei in blue. Scale bar, 10 μm. Red-positive punctate structures were counted in order to quantify the levels of WPBs. NT indicates not treated cells. Values reported for vWF positive structures are the mean ± SD of 3 independent experiments with similar results. Statistical analysis was performed by one-way ANOVA, and the Bonferroni post-test was used to compare data (* *p* < 0.05, ** *p* < 0.01, *** *p* < 0.001). (**B**) HUVECs were serum starved for 16 h. After serum starvation, cells were treated or not for 1 h with the PI3K inhibitor LY294002 (10 μM) or wortmannin (WT [100 nM]) before stimulation or not with 10 ng/mL of p17. Supernatants were collected at 15 and 30 min of culture and analyzed for the presence of vWF by a standard quantitative ELISA. Bars represent the mean ± SD of triplicate samples. Statistical analysis was performed by one-way ANOVA, and Bonferroni post-test was used to compare data (*** *p* < 0.001). NT indicates not treated cells.

PI3Ks are a family of lipid kinases, divided into three classes in mammals, that phosphorylate intracellular inositol lipids to regulate signaling and intracellular vesicular traffic. Class II and III PI3Ks generate phosphatidylinositol 3-phosphate (PI3P) [37], thus functioning as positive regulators for the initiation and progression of autophagy [38]. Indeed, PI3P acts as a signaling molecule and platform for the assembly and coordination of various effector proteins during the autophagic process [39]. Wortmannin and LY294002 are among the early-discovered pan-PI3K inhibitors and are commonly used to block autophagy [40]. In particular, wortmannin is a preferred autophagy inhibitor based on its persistent inhibition of class III PI3K [34]. In order to delineate the function of PI3K in p17-induced vWF accumulation and secretion, HUVECs were incubated with p17 and optimal concentrations of wortmannin or LY294002 [26,32]. As shown in Figure 6A, p17-induced vWF accumulation was significantly inhibited by wortmannin and LY294002. The same inhibitors were able to significantly reduce vWF secretion starting from 15 to 30 min of p17 treatment (Figure 6B). Our results suggest that p17-induced vWF intracellular accumulation and secretion from HUVECs is dependent on activation of the PI3K pathway.

### 2.5. Thrombin Antithrombin (TAT) Complex Formation in Wild Type and Autophagy Deficient Mice Treated With p17

**Figure 7:**
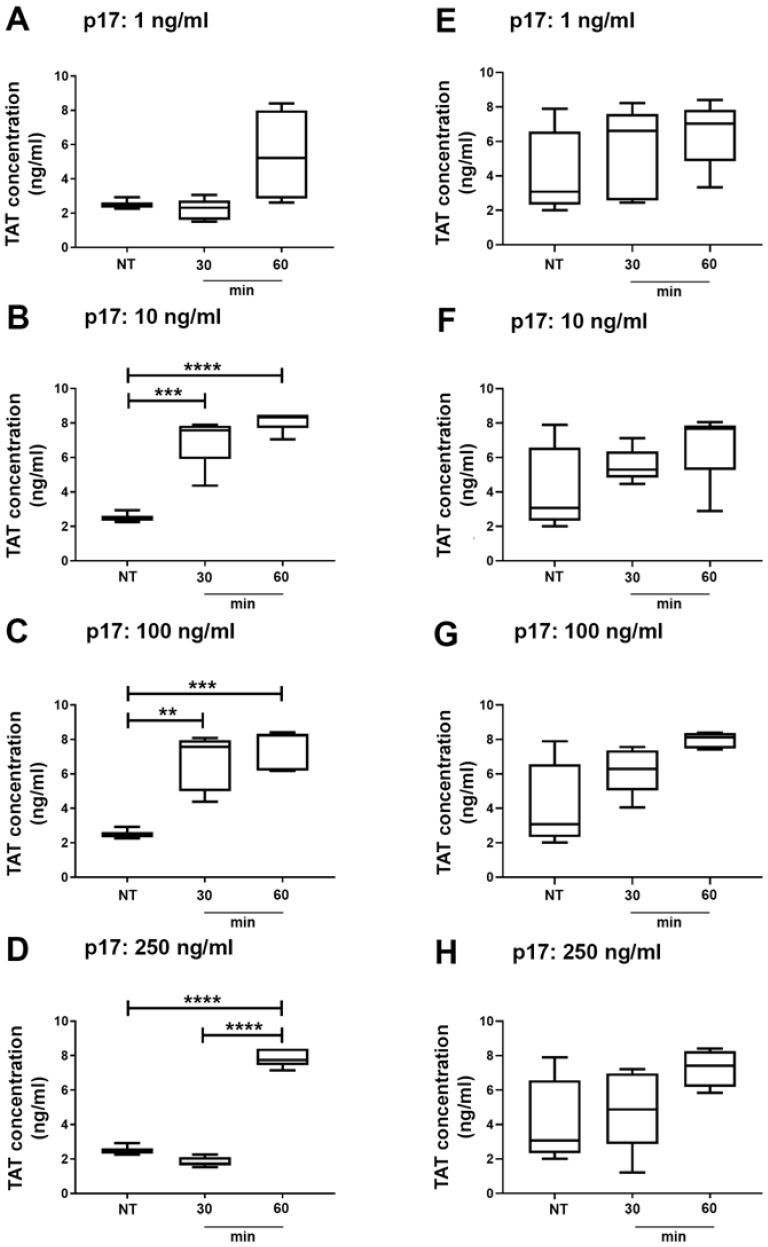
Increased TAT complex formation is observed in plasma from wild type but not autophagy-deficient mice treated with p17. C57BL/6 (**A**–**D**) and BECN-1 (**E**–**H**) mice were i.v. injected or not (NT, not treated mice) with 1 ng/mL (**A**,**E**), 10 ng/mL (**B**,**F**), 100 ng/mL (**C**,**G**) and 250 ng/mL (**D**,**H**) of p17, respectively. Blood samples were collected at 30 and 60 min following p17 injection and further analyzed for TAT complex formation by ELISA. Results are expressed as TAT concentration in plasma of mice. Statistical analysis was performed by one-way ANOVA with Tukey’s multiple comparison test (** *p* < 0.01, *** *p* < 0.001, **** *p* < 0.0001). In box and whiskers graphs, boxes extend from the 25th to the 75th percentiles, lines indicate the median values, and whiskers indicate the range of values. Each box represents the mean ± SEM of five animals.

In order to discover whether we had an autophagy-dependent procoagulant activity triggered by p17 in vivo, we investigated the effect of p17 on coagulation processes in autophagy-deficient (Beclin-1 deficient: BECN-1) mice and in their normal counterpart (C57BL/6). Blood samples were collected at 30 and 60 min after i.v. p17 injection and analyzed for levels of TAT complexes, which measure the intravascular generation of thrombin. As shown in Figure 7 (panels B to D), statistically significant increased levels of TAT complexes were observed in C57BL/6 mice treated with 10, 100 and 250 ng/mL of p17 as compared to not treated (NT) mice. In contrast, no statistically significant increase in TAT levels was observed in autophagy-deficient BECN-1 mice (Figure 7, panels E to H) at any p17 tested dose. Our findings suggest that p17 promotes a rapid thrombin formation and that the p17 activity depends on an intact autophagy pathway.

## 3. Discussion

Our data show that p17 is capable of inducing WPB storage of vWF and its secretion from both macrovascular and microvascular ECs under serum-starvation only. In fact, p17 did not exert any activity on the same ECs grown under normal culture conditions. It is well known that certain stresses (i.e., serum starvation) can induce cells to release autophagic vacuoles [41,42] and, indeed, silencing of either Beclin-1, GRASP55 and Rab8a resulted in reduced WPB storage and secretion of vWF promoted by p17 stimulation. This demonstrates that vWF secretion in p17-treated ECs is dependent on the activation of an autophagy-based pathway. Our data agree with previous studies showing the crucial role of GRASP55 and Rab8a in the unconventional secretion of different cytosolic proteins [1,2,3,36].

The PI3K signaling pathway was previously found to be involved in p17-induced lymphangiogenesis [32] representing a key event for triggering ET-1 secretion from endothelium upon the viral protein stimulation [32]. In this study, we show that p17 triggers vWF release by activating PI3K in starved ECs. Generally, class I PI3Ks are not involved in autophagic processes, being more dedicated to generating 3-phosphoinositide lipids, which directly activate signal transduction pathways. Instead, class II and III PI3Ks are required for the initiation and progression of autophagy. Our data show that 3-MA as well as wortmannin and LY294002 do inhibit the p17-driven vWF accumulation and secretion. 3-MA, similarly to LY294002, exerts dual effects on class I and III PI3Ks, whereas wortmannin is a preferred autophagy inhibitor based on its persistent inhibition of class III PI3K [34]. Since class II are known to be less sensitive to wortmannin than class III PI3K [43], it is likely that the latter coordinates autophagy in starved ECs upon p17 stimulation. However, further studies are needed to better understand the role of class II and/or class III PI3Ks in sustaining p17 activity.

TAT is a molecular complex composed of thrombin and antithrombin, a primary thrombin inhibitor, in a 1:1 ratio [44]. The levels of the complex reflect thrombin generation and it is considered a sensitive marker for the activation of intravascular coagulation [45,46]. To date, increased thrombin levels imply activation of the blood coagulation cascade and have a clinically significant value for the diagnosis and risk assessment of thromboembolic events [47,48]. In vivo data, obtained following i.v. injection of p17 at a concentration as low as 10 ng/mice, showed a rapid and dramatic increase of TAT complex formation, as compared to untreated mice. This finding attests for a potent procoagulant activity of p17 in vivo.

At the same time, Beclin-1 deficient mice were completely unsensitive to the pro-coagulant p17 activity, suggesting that in vivo thrombin formation following p17 injection depends on an intact autophagy pathway. Notably, we have previously demonstrated the role of autophagy in p17-driven ET-1 secretion and angiogenesis [33]. Similarly, impaired granular exocytosis has been observed after inhibition of autophagy within different cells [5,49,50]. Our current results, in conjunction with these previous observations, enforce the role of autophagy in regulating secretion in specialized cell types as ECs.

The spectrum of HIV-1-related clinical disease risk in successfully cART-treated HIV^+^ patients now includes cardiovascular diseases and, in particular, thrombotic events that have been related to persistent aberrations in coagulation [15,16,51,52]. In order to improve therapy and supportive care in HIV^+^ patients, and definitively reduce mortality in the cART era, a better understanding of the pathogenesis of thrombotic complications is needed. The evidence that successfully cART-treated patients display a hypercoagulable state suggests diverse pathogenic mechanisms at work in addition to the direct effects of the HIV-1 itself. The presence of p17 in blood at nM concentration and its capability, at the same concentration, to promote vWF secretion in vitro and TAT complexes in vivo support the hypothesis of a possible role of the viral protein in promoting a pro-coagulative state in HIV^+^ patients even under successful cART.

However, one must also consider that the amount of circulating p17 is likely to differ among HIV^+^ patients, making them more or less prone to develop coagulopathy and other vascular diseases. In addition, other potential drivers of coagulation, such as low level HIV-1 replication in tissues [53], other HIV-1-encoded proteins, co-pathogens as cytomegalovirus [54] and microbial translocation [55], have to be taken into consideration, together with traditional coagulation risk factors including smoking, dyslipidemia, hypertension and diabetes, commonly found in the HIV^+^ population when assessing the thrombotic risk in these patients [56,57]. Long-term studies with clinical endpoints that include careful measurement of circulating p17 levels, together with the presence or absence of neutralizing p17 antibodies, will enable clinicians to interpret research data and place them into a perspective.

## 4. Materials and Methods

### 4.1. Recombinant Proteins and mAb to p17

Purified endotoxin-free recombinant p17 (from clone BH10 of clade B isolate), p24 and GST were produced as previously described [33]. The p17-neutralizing mAb MBS-3 [29] was produced in our laboratory.

### 4.2. Cell Cultures

HUVECs were isolated, characterized and cultured as previously described [58]. HMVEC-Ls were purchased from Clonetics (San Diego, CA, USA) and cultured in EGM-2MV (Lonza, Milan, Italy) containing 10% FBS.

### 4.3. Fluorescent vWF Expression and Quantification

ECs were nucleofected with a mCherry-vWF expressing plasmid [5] by using the Amaxa Nucleofector Technology (Lonza, Milan, Italy). Twenty-four h after nucleofection, cells were serum starved or not for 16 h in endothelial basal medium (EBM) containing 0.5% FBS alone or in combination with 3-MA (5 mM) (Sigma-Aldrich, St. Louis, MO, USA) or a pharmacological inhibitor of cathepsin-β, CA-074Me (50 µM) (Enzo life sciences, Farmingdale, NY, USA). Cells were treated for 1 h with 10 ng/mL of GST, p24 or p17 and fixed with 4% paraformaldehyde. When reported, p17 was preincubated for 30 min at 37 °C with 1 μg/mL of unrelated control mAb (Ctrl mAb) or p17-neutralizing mAb MBS-3. When indicated, serum-starved ECs were treated for 1 h with the PI3K inhibitor LY294002 (10 μM) or WT (100 nM) (Enzo life sciences, Farmingdale, NY, USA) before p17 stimulation. Fluorescence was analyzed using a Leica (Wetzlar, Germany) TCS SP5 laser scanning fluorescence microscope and the imaging software Leica Application Suite. The number of puncta per cell was quantified using ImageJ software, by counting vWF-positive puncta in 20 cells/experiment. Error bars represent the standard deviation calculated as the mean of 3 independent experiments with similar results.

### 4.4. Silencing of Autophagy Related Genes by siRNA Technique

ECs were nucleofected with 300 nM of siRNA. Silencing was carried out using a Beclin-1 siRNA (Cell Signaling Technology, Danvers, MA, USA) and four distinct siRNAs targeting four different regions of Rab8a and GRASP55 (Dharmacon, Lafayette, CO, USA). siScramble (Cell Signaling Technology, Danvers, MA, USA) was used as a negative control.

### 4.5. Human vWF Quantitative ELISA

ECs were serum starved for 16 h and then cultured in the presence of 10 ng/mL of p17. Conditioned medium was collected at 15 and 30 min after the beginning of culture. vWF was measured by ELISA (Thermo Fisher, Waltham, MA, USA). In some experiments, ECs were nucleofected with siScramble, siBeclin-1, siGRASP55 or siRab8a and 24 h after nucleofection, cells were serum starved for 16 h and then stimulated with 10 ng/mL of p17. When reported, ECs were serum starved for 16 h in the presence or absence of 3-MA (5 mM) or CA-074Me (50 μM). In some experiments, 16 h serum-starved ECs were treated for 1 h with PI3K inhibitor LY294002 (10 μM) or WT (100 nM) before p17 stimulation.

### 4.6. Quantification of Murine TAT Complexes by ELISA

Experiments on mice have been ethically approved by the government of Lower Saxony State of Germany with the No. 33.19-42502-04-16/2109. C57BL/6 and BECN-1 mice (carrying a monoallelic deletion of the autophagy-related Beclin-1 gene) (Jackson Laboratory, Bar Harbor, ME, USA), 8–10 weeks old female mice were i.v. injected or not with 1, 10, 100 or 250 ng/mL of p17/mouse (*n* = 5/groups). Citrated plasma samples were collected at 30 and 60 min following p17 injection and analyzed for the presence of TAT complexes by ELISA (Assaypro LLC., St. Charles, MO, USA). Results were expressed as concentration of TAT compared to values of not treated (NT) animals.

### 4.7. Statistical Analysis

Data obtained from multiple independent experiments are expressed as the mean ± the standard deviation (SD) or standard error of mean (SEM). Data were analyzed for statistical significance using one-way analysis of variance (ANOVA). Bonferroni’s post-test or Tukey’s post hoc test were performed. Differences were considered significant at a *p*-value of < 0.05. Statistical tests were carried out using Prism software (GraphPad).

## Supplementary Materials

Supplementary materials can be found at https://www.mdpi.com/1422-0067/21/6/2022/s1. Supplementary Figure S1. The HIV-1 matrix protein p17 induces vWF accumulation in WPBs under serum deprivation. HUVECs were nucleofected with a mCherry-vWF-expressing plasmid and 24 h after nucleofection cells were starved for 16 h and then stimulated in the presence or absence of 10 ng/mL of GST, p24 or p17 in complete medium. The images display vWF signals in red and cell nuclei in blue. Scale bar, 100 μm. NT indicates not treated cells.

## Abbreviations

**3-MA**	3-methyladenine
**AIDS**	acquired immune deficiency syndrome
**cART**	combined antiretroviral therapy
**ECs**	endothelial cells
**ET-1**	endothelin-1
**GRASP**	Golgi reassembly stacking protein
**GST**	glutathione S-transferase
**HIV-1**	human immunodeficiency virus type 1
**p24**	HIV-1 capsid protein p24
**HMVEC-L**	human lung microvascular endothelial cells
**HUVEC**	human umbilical vein endothelial cells
**mAb**	monoclonal antibody
**PI3K**	phosphatidylinositol 3-kinase
**Rab8**	Ras-related protein 8
**TAT**	thrombin anti-thrombin
**vWF**	von Willebrand factor
**WPBs**	Weibel-Palade bodies
