# Supplementary material for: Role of Autophagy in Von Willebrand Factor Secretion by Endothelial Cells and in the In Vivo Thrombin-Antithrombin Complex Formation Promoted by the HIV-1 Matrix Protein p17
Source: Int J Mol Sci. 2020 Mar 16;21(6):2022. doi: 10.3390/ijms21062022 (PMC7139864; doi:10.3390/ijms21062022)
Supplement: Supplementary file 1 [file ijms-21-02022-s001.pdf]

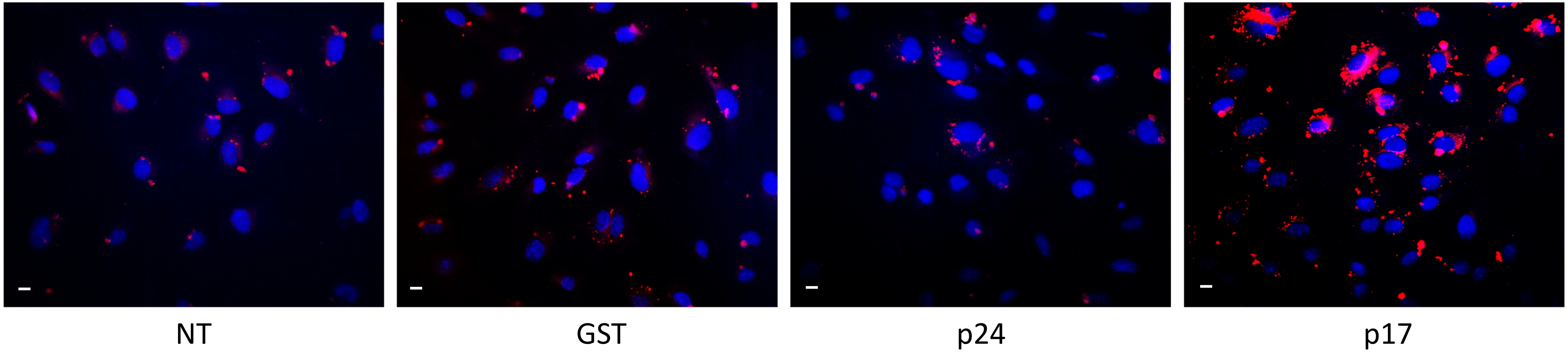

Supplementary Figure 1. The HIV-1 matrix protein p17 induces vWF accumulation in WPBs under serum deprivation. HUVECs were nucleofected with a mCherry-vWF-expressing plasmid and 24 h after nucleofection cells were starved for 16 h and then stimulated in the presence or absence of 10 ng/ml of GST, p24 or p17 in complete medium. The images display vWF signals in red and cell nuclei in blue. Scale bar, 100 μm. NT indicates not treated cells.
